# Amelioration of ovalbumin-induced lung inflammation in a mouse model by *Trichinella spiralis* novel cystatin

**DOI:** 10.14202/vetworld.2023.2366-2373

**Published:** 2023-11-27

**Authors:** Nipa Thammasonthijarern, Kobporn Boonnak, Onrapak Reamtong, Thanyaluk Krasae, Janyaporn Thankansakul, Wallaya Phongphaew, Sumate Ampawong, Poom Adisakwattana

**Affiliations:** 1 Department of Parasitology, Faculty of Veterinary Medicine, Kasetsart University, Bangkok, Thailand; 2 Department of Immunology, Faculty of Medicine Siriraj Hospital, Mahidol University, Bangkok, Thailand; 3 Department of Molecular Tropical Medicine and Genetics, Faculty of Tropical Medicine, Mahidol University, Bangkok, Thailand; 4 Laboratory Animal Science Unit, Faculty of Tropical Medicine, Mahidol University, Bangkok, Thailand; 5 Kasetsart University Veterinary Teaching Hospital, Faculty of Veterinary Medicine, Kasetsart University, Bangkok, Thailand; 6 Department of Pathology, Faculty of Veterinary Medicine, Kasetsart University, Bangkok, Thailand; 7 Department of Tropical Pathology, Faculty of Tropical Medicine, Mahidol University, Bangkok, Thailand; 8 Department of Helminthology, Faculty of Tropical Medicine, Mahidol University, Bangkok, Thailand

**Keywords:** asthma, immunomodulatory molecule, recombinant *Trichinella spiralis* novel cystatin, *Trichinella spiralis*

## Abstract

**Background and Aims:**

Asthma, a chronic disease affecting humans and animals, has recently become increasingly prevalent and steadily widespread. The alternative treatment of asthma using helminth infections or helminth-derived immunomodulatory molecules (IMs) has been evaluated and demonstrated significant amelioration of disease severity index *in vitro* and *in vivo*. *Trichinella spiralis*, a parasitic nematode and its IMs, elicits a potential to relieve asthma and other immune-related disorders. In this study, we investigated the immunomodulatory function of recombinant *T. spiralis* novel cystatin (rTsCstN) in ameliorating acute inflammatory asthma disorders in a murine model.

**Materials and Methods:**

Female BALB/c mice were sensitized using intraperitoneal injection of ovalbumin (OVA)/alum and subsequently challenged with intranasal administration of OVA alone or OVA + rTsCstN for 3 consecutive days, producing OVA-induced allergic asthma models. To evaluate the therapeutic efficacy of rTsCstN, the inflammatory cells and cytokines in bronchoalveolar lavage fluid (BALF) and OVA-specific immunoglobulin E levels in serum were assessed. Histological alterations in the lung tissues were determined by hematoxylin and eosin (H&E) staining and eventually scored for the extent of inflammatory cell infiltration.

**Results:**

The asthmatic mouse models challenged with OVA + rTsCstN demonstrated a significant reduction of eosinophils (p < 0.01), macrophages (p < 0.05), and cytokines tumor necrosis factor-α (p < 0.05) and interferon (IFN)-γ (p < 0.05) in BALF when compared with the mice challenged with OVA alone. However, the levels of interleukin (IL)-4 and IL-10 remained unchanged. Histological examination revealed that mice administered OVA + rTsCstN were less likely to have inflammatory cell infiltration in their perivascular and peribronchial lung tissues than those administered OVA alone.

**Conclusion:**

Recombinant *T. spiralis* novel cystatin demonstrated immunomodulatory effects to reduce severe pathogenic alterations in asthma mouse models, encouraging a viable alternative treatment for asthma and other immunoregulatory disorders in humans and animals in the future.

## Introduction

Asthma, a chronic inflammatory respiratory tract condition, is primarily triggered by exposure to environmental pollutants *such as* particulate matter 2.5 and allergens, *e.g*., house dust mites (HDM) [1–4]. The pathogenesis of asthma involves airway inflammation induced by T helper 2 (Th2), resulting in obstruction, mucus overproduction, and airway wall remodeling [2, 5–8]. Elevated levels of the cytokines interleukin (IL)-4, IL-5, and IL-13 are associated with Th2-mediated eosinophilic disorders [6, 9, 10]. The clinical signs of allergic asthma include wheezing, coughing, shortness of breath or difficulty breathing, and limited airflow [1, 5]. Due to its severity, lack of treatment, medication, or vaccine, allergic asthma poses a major threat to human and animal health. Recently, in wealthy developed countries, the prevalence of asthma has increased, coinciding with the near eradication of helminthic infections. The lack of exposure to helminths is believed to be one of the factors contributing to immune dysregulation and, thereby, increased vulnerability to immune-mediated inflammatory disorders (IMIDs) such as inflammatory bowel disease, multiple sclerosis, type 1 diabetes, and rheumatoid arthritis [6, 11]. Helminthic infections exert immunomodulatory effects by secreting proteins or substances that suppress host immune responses, which benefit parasite survival [10, 12–16]. In IMIDs, cytokine milieu imbalance and aberrant or excessive immune cell responses lead to acute and chronic inflammation [17]. According to the immunomodulation of helminthic infections, helminths or their immunomodulatory molecules (IMs) have been employed in several studies and clinical trials to treat IMIDs in animal models and human patients [10, 13, 17]. In allergic asthma, the infections with helminths or treatment with helminth-derived molecules diminished disease morbidity in animal models and humans [6, 10, 18]. Various helminths, such as filarial [19, 20], *Ascaris lumbricoides* [16, 21, 22], *Schistosoma* spp. [23, 24], *Haemonchus contortus* [25] as well as *Trichinella spiralis* [8, 11, 26–28] and their IMs, have been identified to possess anti-inflammatory properties against asthma.

*Trichinella spiralis*, a tissue-dwelling helminth that causes trichinellosis, is a prominent global parasitic nematode that infects humans and other mammalian species. To ensure their long-term survival within the host, these parasites employ immune evasion mechanisms such as intracellular infection, nurse cell production, and secretion of IMs [8, 11, 29]. Recent investigations have revealed that infection with *T. spiralis* or treatment with *T. spiralis* extracts can reduce allergic inflammation in experimental asthma mouse models [8, 30]. In our previous study, we identified a novel cystatin called TsCstN in the excretory-secretory (ES) products of *T. spiralis* ES-L1 that suppressed macrophage inflammation by decreasing major histocompatibility complex class II expression and production of proinflammatory cytokines [29]. Similarly, a filarial cystatin, namely Av17, decreased Th2-mediated inflammation and asthma-related symptoms of ovalbumin (OVA)-induced allergic airway hyper-responsiveness (AHR) in an asthma mouse model [31]. Furthermore, a HDM-induced AHR in a mouse model was treated with *A. lumbricoides* recombinant cysteine protease inhibitor (rAl-CPI), which substantially reduced goblet cell hyperplasia, eosinophil/neutrophil infiltration in perivascular/peribronchial regions, and levels of Th2 cytokines [21].

This study aimed to assess the potential of recombinant *T. spiralis* novel cystatin (rTsCstN) in suppressing OVA-specific Th2 responses in an asthma mouse model based on the immunomodulatory properties exhibited by helminth cystatins in reducing airway inflammation.

## Materials and Methods

### Ethical approval

All experimental procedures and housing conditions were performed on mice according to the Ethical Principles and Guidelines for the Use of Animals of the National Research Council of Thailand. The study was approved by the Faculty of Tropical Medicine Animal Care and Use Committee (FTM-ACUC), of Mahidol University, with approval number FTM-ACUC010/2021.

### Study period and location

This study was conducted from September 2021 to August 2022 in the Animal Care Unit at the Faculty of Tropical Medicine, Mahidol University, Bangkok, Thailand.

### Animals

A total of 15 female BALB/c mice, aged 6–8 weeks, were purchased from Nomura Siam International Company, Bangkok, Thailand. The mice were housed in the Animal Care Unit at the Faculty of Tropical Medicine, Mahidol University, Bangkok, Thailand.

### Expression and purification of rTsCstN

The rTsCstN was produced and purified following the methods described in our previous study by Kobpornchai *et al*. [29]. To facilitate protein refolding, a rTsCstN was dialyzed against 1× phosphate-buffered saline (1× PBS, pH 7.4). The endotoxin contamination in rTsCstN was removed using the Pierce High-Capacity Endotoxin Removal Resin according to established protocols of the manufacturer (Thermo Fisher Scientific Inc., Waltham, MA). The residual endotoxin level (<0.5 EU/mL) was measured using the Pierce LAL Chromogenic Endotoxin Quantitation kit (Thermo Fisher Scientific, Waltham, USA) and had to be <0.5 EU/mL to meet the endotoxin regulatory standard established by the US Food and Drug Administration.

### Ovalbumin sensitization and treatment

**Figure-1:**
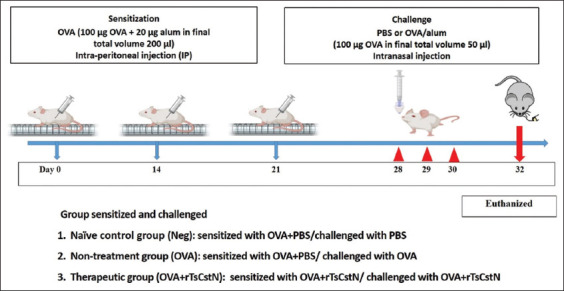
Experimental schedule of allergic asthma model for OVA sensitization/challenge or rTsCstN treatment.

Five female (6–8 weeks old) BALB/c mice in each group (a total of three groups) were sensitized three times (day 0, 14, and 21) by intraperitoneal injection of 100 μg of OVA (Sigma-Aldrich, Steinheim, Germany) emulsified in 20 μg of alum (Imject Alum, Thermo Fisher Scientific) in a total volume of 200 μL. For therapeutic groups, mice were co-administered intraperitoneally with 50 μg of rTsCstN during the OVA sensitization on days 0, 14, and 21. One week after the last sensitization (on days 28, 29, and 30), all mice were anesthetized with isoflurane and intranasally challenged with 100 μg of OVA in a total volume of 50 μL of sterile 1× PBS. In addition, the mice in the therapeutic group received intranasal co-administration of 25 μg of rTsCstN during the OVA challenge (Figure-1). Mice sensitized and challenged with OVA alone were used as the non-treatment group. Moreover, a group of mice injected with alum in PBS and mock-challenged using PBS served as the naïve group (Neg). All mice were euthanized 48 h after the last challenge, and bronchoalveolar lavage fluid (BALF) and sera were collected immediately to determine immune cell infiltration, cytokine level, and immunoglobulin E (IgE) level, respectively. Lung tissue samples were collected for histopathological analysis and all experiments were performed in duplicate.

### Bronchoalveolar lavage

Following euthanasia, the lung airways of the mice were lavaged four times with 0.7 mL of sterile 1× PBS. The lavage fluid was then centrifuged at 1500× *g* for 10 min at 4°C, and the resulting supernatants were collected and stored at −80°C for subsequent cytokine measurements. Subsequently, the cells present in the sediment were analyzed using FACSCalibur (BD) to identify CD45^+^ cells, alveolar macrophages (Siglec-F^+^CD11c^+^), eosinophils (Siglec-F^+^CD11c^−^), and neutrophils (Siglec-F^−^CD11b^+^Ly6G^+^) [21]. Data were analyzed using FlowJo software version 10 (TreeStar Inc., USA). The fluorochrome-conjugated monoclonal antibodies were used for flow cytometry and the gating strategy was used to identify inflammation cells in BALF. (Supplementary data).

### Measurement of cytokines in BALF and OVA-specific IgE levels in sera

The concentrations of IL-4, IL-10, tumor necrosis factor (TNF)-α, and interferon (IFN)-γ in BALF were measured using enzyme-linked immunosorbent assay (ELISA) MAX™ Deluxe Set (BioLegend, San Diego, CA) according to the manufacturer’s instructions. The optical density values were measured at 450 nm and 570 nm using an ELISA plate reader (Synergy H1 Hybrid Reader, BioTek, Winooski, VT). The blood samples were obtained from the submandibular vein (cheek pouch) and allowed to clot at room temperature (25°C) until clotting. The sera were collected by centrifugation at 2,000× *g* for 10 min at 4°C. The levels of OVA-specific IgE were determined using the Mouse IgE ELISA MAX™ Standard (BioLegend) according to the manufacturer’s recommendations.

### Histopathological analysis

Lung tissue samples were fixed in 10% neutral-buffered formalin and embedded in paraffin. Sections were cut and subjected to hematoxylin and eosin staining. Slides of lung tissue sections were observed under a light microscope (Olympus Corporation, Tokyo, Japan), and the extent of cell infiltration around the basal membrane of bronchi or vessels was assessed. Lung inflammation severity was graded on a scale of 0–4 according to the following criteria: 0: normal cells, 1: few cells inflammations in 1 cell layer deep, 2: thin layer of inflammatory cells infiltration (two to three cells thick); 3: thick layer of four to five inflammatory cells surrounding, and 4: bronchi or vessels surrounded by a layer of more than five inflammatory cells [1].

### Statistical analysis

Statistical analysis was performed using GraphPad Prism 6.0 software (GraphPad Software Inc., San Diego, CA, USA). All data are presented as mean values with standard deviation (Mean ± SD) unless otherwise indicated. To determine differences among three or more groups, a one-way analysis of variance followed by Tukey’s test for multiple comparisons was performed.

## Results

### Mice treated with TsCstN reduced eosinophils recruitment in BALF

**Figure-2:**
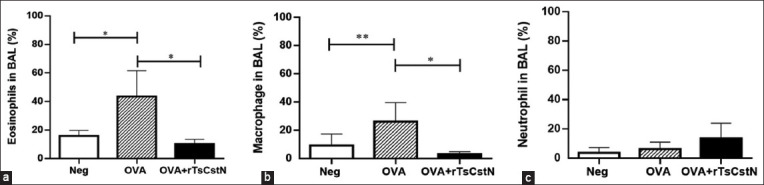
Treatment of OVA-sensitized and challenged mice with rTsCstN (OVA + TsCstN) significantly reduced (a) eosinophil (b) and macrophage cells infiltrated into BALF when compared with the mice sensitized and challenged with OVA alone. (c) Nonetheless, the neutrophil number did not change in any of the three groups. Mice sensitized with OVA + PBS and challenged with PBS were used as naïve control group (Neg). The results are expressed as mean ± SD. The experiments (n = 5 mice per group) were performed as two independent experiments. One-way ANOVA followed by the Tukey’s multiple comparison test was employed for analysis. *p < 0.01, **p < 0.05.

Eosinophils, macrophages, and neutrophil levels in BALF of OVA-sensitized and challenged mice were assessed. When compared with naïve animals sensitized with OVA + PBS/challenge with PBS (Neg), the results revealed a significant increase (p ≤ 0.001) in the numbers of eosinophils and macrophages in the BALF of OVA-sensitized and challenged mice. Interestingly, the treatment with rTsCstN significantly reduced the infiltration of eosinophils and macrophages in the BALF in OVA-sensitized and challenged mice (OVA + rTsCstN) (p ≤ 0.01) (Figures-2a and b). However, no significant difference was observed in the number of neutrophils among the three groups (Figure-2c). The flow cytometry results are provided in Supplementary data.

### Recombinant *T. spiralis* novel cystatin reduced inflammatory cells in lung parenchyma of OVA-sensitized and challenged mice

**Figure-3:**
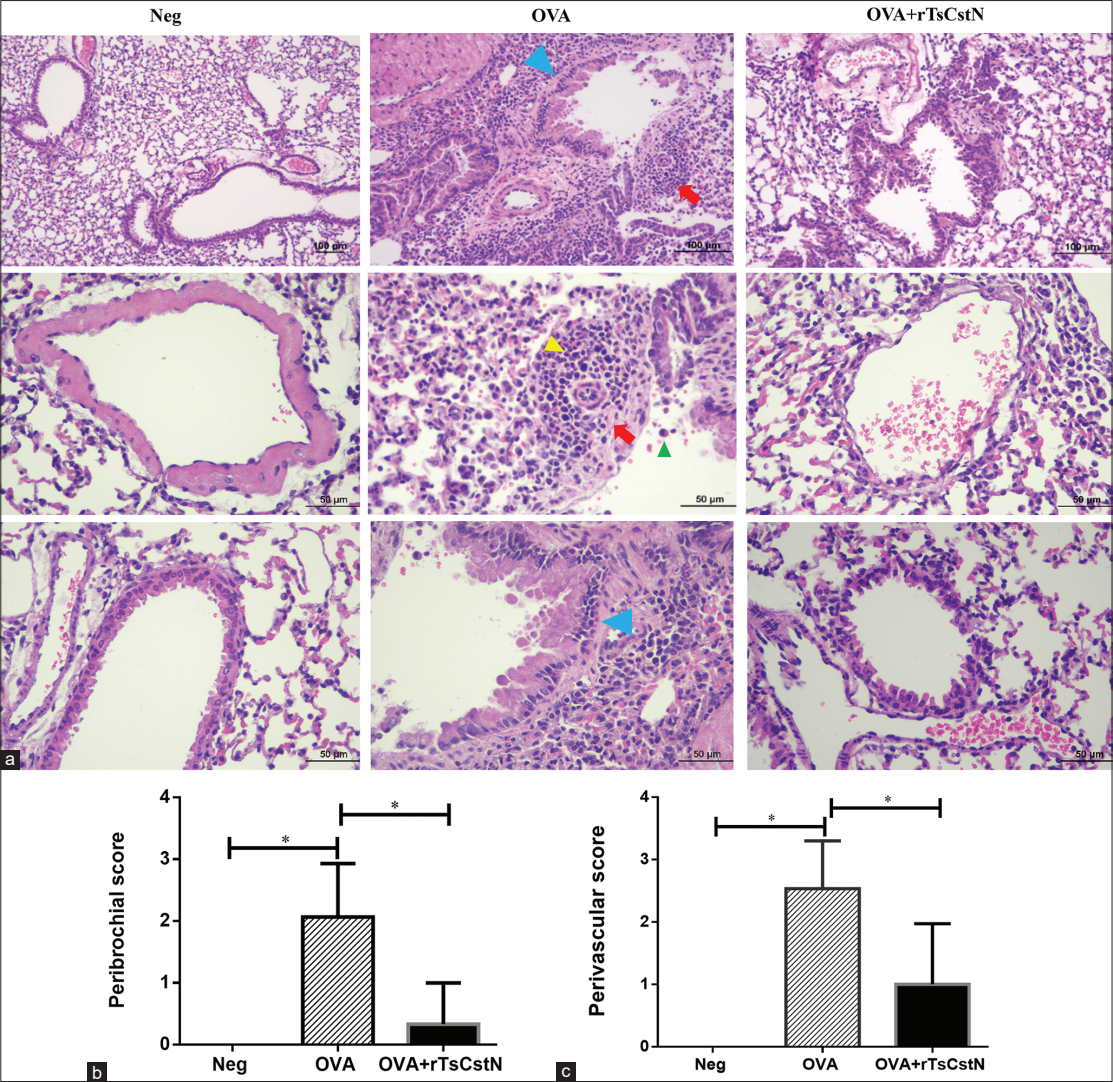
Histopathological changes of lung tissues (a) of OVA-sensitized/challenged mice with or without treatment of TsCstN; (Upper) low-power field (×200) of lung parenchyma, (Middle) high-power field (×400) of perivascular cuffing (red arrow) infiltrated with eosinophils (yellow arrowhead) and lung macrophage (green arrowhead), (Lower) high-power field (×400) of peribronchial cuffing, blue arrow. The inflammatory cells infiltration score was determined and it was observed that the treatment of OVA-sensitized/challenged mice with rTsCstN regressed the cell infiltration score at (b) peribronchial area and (c) perivascular area. The results are expressed as mean ± SD. Neg: naïve mice sensitized with OVA + PBS and challenged with PBS, OVA: mice sensitized and challenged with OVA alone, OVA + rTsCstN: mice sensitized with OVA + rTsCstN and challenged with OVA + rTsCstN. The experiments (n = 5 mice per group) were performed as two independent experiments. One-way ANOVA followed by Tukey’s multiple comparison test was used for analysis. *p < 0.01.

Mice sensitized and challenged with OVA exhibited lung pathology resembling asthma, including perivascular and peribronchial cell infiltration, lung edema, and lung hemorrhage [1, 21]. When mice were sensitized and challenged with OVA alone, it induced lung inflammation, resulting in enormous infiltration of immune cells, especially eosinophils in the lung parenchyma, particularly in the perivascular and peribronchial areas (Figure-3a). Conversely, naïve mice sensitized with OVA + PBS and challenged with PBS (Neg) did not develop lung inflammation and cell infiltration in the lung parenchyma. However, the administration of rTsCstN to OVA-sensitized/challenged mice (OVA + rTsCstN) reduced lung inflammation and cell infiltration, as evidenced by a substantial decrease in peribronchial and perivascular scores (Figures-3b and c).

### Treatment of OVA-sensitized/challenged mice with rTsCstN inhibited proinflammatory cytokines

**Figure-4:**
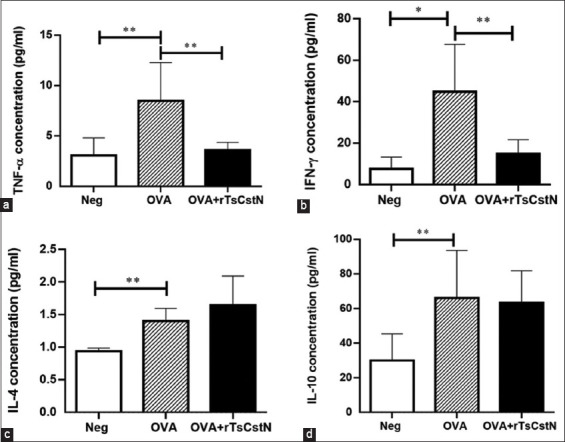
Impact of rTsCstN treatment on the cytokine responses in an experimental lung allergy model. The cytokine levels of (a) TNF-α, (b) IFN-γ, (c) IL-4, and (d) IL-10 were measured in BALF of OVA-sensitized mice upon treatment with rTsCstN. The mice sensitized with OVA + PBS/challenge with PBS were used as naïve control group (Neg). Data are expressed as mean ± SD from two independent experiments (n = 5 mice per group). One-way ANOVA followed by Tukey’s multiple comparison test was used for analysis. *p < 0.01, **p < 0.05.

In addition to immune cell infiltration into BALF and lung tissue, local cytokines, including Th1-(TNF-α and IFN-γ), Th2-(IL-4), and regulatory cytokines (IL-10) in BALF of these mice were analyzed. Mice sensitized and challenged with OVA alone exhibited significantly higher levels of TNF-α, IL-4, and IL-10 as compared with the naïve control group (Neg) (p ≤ 0.05) (Figures-4a-d). However, when comparing the OVA- and Neg groups, the level of IFN-γ in BALF was significantly increased (p ≤ 0.01) (Figure-4b). Treatment of OVA-sensitized/challenged mice with rTsCstN (OVA + rTsCstN) significantly reduced the levels of proinflammatory cytokines, including TNF-α and IFN-γ, in the BALF as compared with the Neg group (p ≤ 0.05) (Figures-4a and b). However, the treatment with rTsCstN did not alter the IL-4 and IL-10 levels in mice that were sensitized and challenged with OVA (p > 0.05 and p > 0.05, respectively) (Figures-4c and d).

### Recombinant *T. spiralis* novel cystatin treatment reduced the level of specific IgE against OVA in the serum of a lung-allergic mouse model

**Figure-5:**
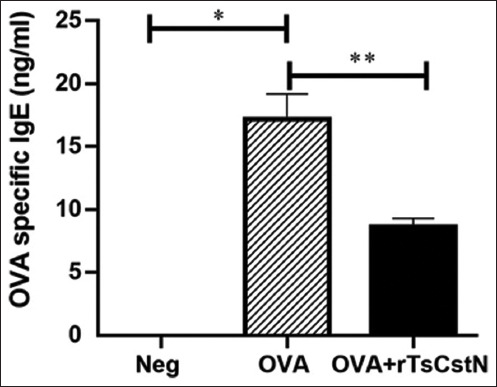
Level of serum OVA-specific IgE significantly increases in mice sensitized and challenged with OVA alone, compared with naïve control mice (Neg). Treatment of OVA-sensitized/challenged mice with rTsCstN (OVA + rTsCstN) decreased the level of OVA-specific IgE. Data are expressed as mean ± SD from two independent experiments (n = 5 mice per group). One-way ANOVA followed by Tukey’s multiple comparison test was used for analysis. *p < 0.01, **p < 0.05.

The level of serum OVA-specific IgE was significantly higher in the OVA-sensitized/challenged group when compared with the naïve control group (Neg). However, when OVA-sensitized/challenged mice were treated with rTsCstN, serum-specific IgE levels significantly decreased compared with those in the OVA group (Figure-5).

## Discussion

Since a decade, allergic asthma has become a growing problem in humans as well as animals, necessitating a novel, effective treatment. To date, several helminth-derived molecules such as proteins, RNA, lipids, and small organic molecules have shown promising therapeutic effects in animal models of asthma, providing evidence to promote clinical trials [8, 21, 32]. Among these molecules, the cysteine protease inhibitors or cystatins released by parasitic helminths have emerged as promising therapeutic agents for treating patients with allergic diseases triggered by allergen exposure [17, 33]. Cystatins have been identified as significant immune modulators associated with nematode parasite infections [17]. They primarily target monocytes and macrophages for immunomodulation [17, 34]. For instance, cystatin produced by *Acanthocheilonema viteae* can stimulate macrophages to produce a considerable amount of IL-10, which reduces clinical damage in mouse models of OVA-induced allergic asthma and DSS-induced colitis [31, 35]. Furthermore, cystatins from *Brugia malayi* and *Schistosoma japonicum* stimulate the alternate activation of peritoneal macrophages and regulatory T cells (Treg), thereby decreasing the production of proinflammatory cytokines in colon tissue [17, 34, 36].

For *T. spiralis*, cystatin can block the Th1-type immune response in TNBS-induced colitis by boosting the production of IL-4 and Treg cells [33, 37]. In asthma, the macrophages release proinflammatory cytokines, including TNF and IL-1, which contribute to chronic airway inflammation [38]. Therefore, in this study, we examined the immunomodulatory effects of rTsCstN on a mouse model of allergic airway inflammation induced by OVA.

Our findings in a mouse model revealed that administering rTsCstN during OVA sensitization and challenge significantly decreased the infiltration of inflammatory cells, including eosinophils and macrophages in BALF, which is consistent with other helminth cystatins. *Ascaris lumbricoides* cystatin (rAl-CPI) treatment of house dust mite-induced airway allergy mice reduced the lung inflammatory response, including eosinophils and neutrophils in BALF [21]. In addition, the reduction of inflammatory cell infiltration by filarial cystatin (rAv17), particularly eosinophil in BALF of OVA-induced airway inflammation, was observed [31]. In immunopathology of lung tissues, the treatment with rTsCstN remarkably diminished immune cell infiltrations into the perivascular and peribronchial areas of OVA-sensitized/challenged mice. In allergic asthma, it is associated with eosinophilic inflammation in the airways, and the proinflammatory mediators produced by eosinophils are major contributors to lung inflammation in asthma [39]. As demonstrated in our study, reducing the infiltration of inflammatory cells, particularly eosinophils into the lungs can prevent lung tissue damage in allergic asthma models.

Furthermore, the OVA-sensitized/challenged mice revealed a significant reduction in TNF-α and IFN-γ levels but not IL-4 or IL-10 levels in the BALF after treatment with rTsCstN. Similar to our previous findings, this study demonstrated that rTsCstN significantly decreased the expression of iNOS and synthesis of proinflammatory cytokines such TNF-α, IL-1β, and IFN-γ in an *in vitro* study employing LPS-stimulated mouse bone marrow derived macrophage [29]. The anti-inflammatory properties of TsCstN in animal models were similar to those of other helminthic cystatins such as Al-CPI and Av17. However, neither the prior study (*in vitro*) [25] nor this study (*in vivo*) analysis of rTsCstN showed any evidence of an elevation of the regulating cytokine (IL-10). The absence of the immunoregulatory properties of TsCstN to elevate IL-10 production might be due to the lack of Treg cell-inducing epitope in the classical cystatins (Al-CPI and Av17).

In our previous investigation, we discovered that TsCstN and its homologs share only 23.08%–37.50% amino acid homology [25], suggesting that our novel cystatin regulates immunity through a different mechanism than the classic cystatin. In addition to rTsCstN reducing the number of proinflammatory cytokines in the BALF of OVA-sensitized/challenged mice, the treatment using rTsCstN significantly downregulated the serum OVA-specific IgE levels. In the prior study, proinflammatory cytokines, particularly TNF-α, were found to be essential for the production of antigen-specific IgE, and a deficiency in TNF-α prevented the emergence of allergic rhinitis [40].

## Conclusion

In this study, our findings revealed that administering novel cystatin of *T. spiralis* (rTsCstN) during OVA sensitization and challenge in a mouse model significantly decreased the number of inflammatory cells and proinflammatory cytokines in the BALF. In addition, treatment with rTsCstN significantly downregulated the serum OVA-specific IgE levels and reduced the severity of lung pathological alterations.

In summary, the amelioration of lung inflammation in the allergic asthma model by rTsCstN offers the possibility of using it to develop a novel treatment for allergic asthma. Future research will investigate the specific protein domain of TsCstN that plays a crucial role in inhibiting inflammation, with the aim of developing small-molecule therapeutics.

## Authors’ Contributions

PA: Supervision. NT and PA: Conceptualization, designed the study, data curation, formal analysis, methodology, writing-original draft preparation and editing of the manuscript. OR, KB, TK, JT, WP, and SA: Methodology and editing of the manuscript. All authors have read, reviewed, and approved the final manuscript.
